# Dr. Henry Simpson

**Published:** 1911-04-22

**Authors:** 


Dr. Henry Simpson, late of Bowdon, and Manchester, has died in North Wales, aged eighty-two. In 1851 Dr. Simpson took his M.R.C.S., and the L.S.A. a year later. He studied at London University, where he graduated M.D. in 1861.

